# Rutin alleviates colon lesions and regulates gut microbiota in diabetic mice

**DOI:** 10.1038/s41598-023-31647-z

**Published:** 2023-03-25

**Authors:** Cifeng Cai, Wenwen Cheng, Tiantian Shi, Yueling Liao, Meiliang Zhou, Zhiyong Liao

**Affiliations:** 1 College of Life and Environmental Science, Wenzhou University, Wenzhou, 325035 People’s Republic of China; 2 Institute of Crop Sciences, Chinese Academy of Agricultural Sciences, Beijing, 100081 People’s Republic of China

**Keywords:** Cell biology, Immunology, Microbiology

## Abstract

Diabetes is a common metabolic disorder that has become a major health problem worldwide. In this study, we investigated the role of rutin in attenuating diabetes and preventing diabetes-related colon lesions in mice potentially through regulation of gut microbiota. The rutin from tartary buckwheat as analyzed by HPLC was administered intragastrically to diabetic mice, and then the biochemical parameters, overall community structure and composition of gut microbiota in diabetic mice were assayed. The results showed that rutin lowered serum glucose and improved serum total cholesterol, low-density lipoprotein, high-density lipoprotein, triglyceride concentrations, tumor necrosis factor-α, interleukin-6, and serum insulin in diabetic mice. Notably, rutin obviously alleviated colon lesions in diabetic mice. Moreover, rutin also significantly regulated gut microbiota dysbiosis and enriched beneficial microbiota, such as *Akkermansia* (p < 0.05). Rutin selectively increased short-chain fatty acid producing bacteria, such as *Alistipes* (p < 0.05) and *Roseburia* (p < 0.05), and decreased the abundance of diabetes-related gut microbiota, such as *Escherichia* (p < 0.05) and *Mucispirillum* (p < 0.05). Our data suggested that rutin exerted an antidiabetic effect and alleviated colon lesions in diabetic mice possibly by regulating gut microbiota dysbiosis, which might be a potential mechanism through which rutin alleviates diabetes-related symptoms.

## Introduction

Diabetes, classified as type 1 diabetes mellitus (T1DM) and type 2 diabetes mellitus (T2DM), is a metabolic disorder accompanied by insufficient insulin production or insulin resistance^1^. To date, although there have been some antidiabetic drugs in clinical practice, there is still a general lack of effective approaches to prevent the initiation and development of diabetes and diabetes-related diseases^2^. In T2DM patients, the incidence of colorectal cancer (CRC) is higher than that in nondiabetic populations^3^. Hyperinsulinemia, hyperglycemia and chronic inflammation are common risk factors for CRC and liver cancer^4^. Colon lesions appear in an initial phase of diabetes-induced CRC. Due to the lack of understanding of the CRC development process, there are currently no effective methods to prevent the development of colon lesions. Studies have shown that the incidence of diabetes is closely related to intestinal flora which plays an important role in host metabolism, immune regulation, the development of inflammation-derived disease and chronic inflammation^5–7^. The composition of the gut microbiota is also significantly correlated with obesity and diabetes^8–10^. Although an increasing number of drugs are being used to treat diabetes and related diseases, the side effects of many drugs cannot be ignored^11^. Therefore, it is of great value to develop new effective and nontoxic drugs for the treatment of diabetes and its related diseases. At present, increasing attention has been paid to the development of natural products due to their low cost and minimal side effects in diabetes treatment.

Rutin, a type of flavonoid glycoside, is mainly found in plants, such as apple, black tea, vegetables and buckwheat^12,13^. It was reported that rutin had antioxidant activity and promoted intestinal absorption^14^. Rutin has attracted extensive attention due to its various biological activities, such as antioxidant^15^, antiinflammatory^16,17^, antidiabetic^13,18–22^, and anticancer activities^23–25^. Recent studies have indicated that the regulation of gut microbiota might be one of the potential mechanisms by which natural products achieve antidiabetic effects^26^. However, whether rutin from tartary buckwheat could improve diabetes and colon lesions by modulating gut microbiota is still not clear. Here, we investigated the effect of rutin on reducing the diabetes pathological state, diabetes-related colon lesions, overall community structure and composition of gut microbiota in diabetic mice and their potential relationship. Our discovery of rutin reducing colon lesions and modulating the gut microbiota composition in diabetic mice provided evidence for the therapeutic potential of rutin to treat diabetes-related symptoms possibly by promoting intestinal health.

## Materials and methods

### Chemicals and reagents

Control diet (10% lipids, 19% protein, 71% carbohydrates, Cat. AIN-93) was obtained from Jiangsu-Xietong, Inc, Nanjing, China. A high-fat diet (HFD) (60% fat, Cat. D12492) was from Research Diets Incorporated Company, New Brunswick, New Jersey, USA. Streptozotocin (STZ, Cat. 18883-66-4) and saline (Cat. 231-598-3) were purchased from Sigma-Aldrich Co., Ltd (St. Louis, MO, USA). Tartary buckwheat powder (Zhongku No. 3) was obtained from Weining Dongfang Shengu Co., Ltd. (Guizhou, China). The AB-8 macroporous resins (Cat. M0042) were purchased from Solarbio Technology Ltd (Beijing, China). All other chemical reagents used in this study were of analytical grade.

### Rutin preparation from tartary buckwheat

The rutin used in this study was obtained by ethanol extraction from tartary buckwheat powder, the purity of rutin prepared by us reached more than 96% and stored in our laboratory as previously described^27^.

### Establishment and treatment of the diabetic mouse model

Forty male C57BL/6 J mice (8 weeks old) were obtained from Shanghai Slac Laboratory Animal Co., Ltd (Shanghai, China). All mice were raised in separate cages under conditions in which they could freely ingest available solid feed and water. The mice were caged at a room temperature of 23 ± 1 °C, humidity of 60% and a 12 h light–dark cycle. The mice were provided with ad libitum access to food and water for 1 week. The STZ-induced T1DM mouse model and HFD/STZ-induced T2DM mouse model were established as previously described^28,29^. Briefly, the mice received intraperitoneal injections of STZ (45 mg/kg/day) for five consecutive days. Four weeks after diabetes induction, fasting blood glucose level of 16.7 mM and above were considered diabetic. The HFD/STZ-induced T2DM mouse model were established, the ND group was fed with a normal diet (10% lipids, 19% protein, 71% carbohydrates) sustainably, the other three groups of mice were fed the HFD (45% lipids, 19% proteins and 36% carbohydrates, research diets incorporated company, New Brunswick, New Jersey, USA) for 8 weeks. After 8 weeks of HFD feeding, the mice were fasted for 12 h overnight. The HFD groups were received intraperitoneal injections of STZ (35 mg·kg − 1, dissolved at 0.1 mol/L cold citrate buffer, pH 4.4) daily for three consecutive days. The fasting serum glucose was sampled from the tail vein and determined by an enzymatic colorimetric assay using a modified glucose oxidase–peroxidase method (Roche Diagnostics, Mannheim, Germany) and a glucose analyzer (Roche-Hitachi 917). Diabetes was defined as abdominal serum glucose ≥ 16.7 mmol/L for two consecutive days^30–32^. In a follow-up experiment, the successfully established diabetes mice were used in the experiment, and the failed models were excluded. The dosage of rutin was optimized according to previous reports^33^. Rutin or saline was administered intragastrically in T1DM and T2DM mice groups ( 5 mice per group) once a day for 4 weeks as follows: ND group (normal diet mice with daily administration of saline), T1DM group (T1DM mice with daily administration of saline), T1DM-Rutin100 group (mice with daily administration of 100 mg Rutin/kg body weight), and T1DM-Rutin200 group (mice with daily administration of 200 mg Rutin/kg body weight), T2DM control group (mice with daily administration of normal saline), T2DM-Rutin100 group (mice with daily administration of 200 mg Rutin/kg body weight), T2DM-Rutin200 group (mice with daily administration of 200 mg Rutin/kg body weight). Four weeks after rutin treatment, fresh fecal samples were collected under a sterile environment and frozen at − 80 °C for subsequent analysis. Serum samples were collected and centrifuged at 3000 rpm for 15 min at 4 °C. Colon tissues and intestinal contents were collected and frozen in liquid nitrogen and stored at − 80 °C. All animal experiments were approved by the Wenzhou University Animal Care and Use Committee (Wenzhou, China) with approval number WZU-2020-010. All methods were performed in accordance with the relevant guidelines and regulations. This study is reported in accordance with ARRIVE guidelines (Animal Research: Reporting of in vivo Experiments; https://arriveguidelines.org).

### Biochemical analysis

Commercial detection kits (Jiancheng Bioengineering Institute, Nanjing, China) were used to measure total serum cholesterol (TC) (Cat. A111-1-1), triglyceride (TG) (Cat. A110-1-1), low-density lipoprotein cholesterol (LDL-C) (Cat. A113-1-1) and high-density lipoprotein cholesterol (HDL-C) (Cat. A112-1-1). Commercial detection kits (MultiSciences Biotech Co., Ltd., Hangzhou, China) were used to measure serum TNF-α (Cat. EK282/4-01) and IL-6 (Cat. 70-EK206/3-96) levels. The fasting serum insulin levels were measured using a Mouse INS(Insulin) ELISA Kit (Cat. D721159-0096, Sangon Biotech Co., Ltd., Shanghai, China).

### Haematoxylin & Eosin (H&E) staining

Colon tissues were fixed with 4% paraformaldehyde for 48 h and all samples were dehydrated with ethanol and embedded in paraffin wax. Tissues were sliced into 5 μm-thick Sections (3–5 sections/specimen) and stained with H&E. Samples were sealed with neutral resin and observed under optical microscope (× 400 magnification) for evaluation. The histopathological score of colonic lesions was measured using the following parameters: the degree of inflammation, crypt damage, number of vacuoles and arrangement of villi. The final pathological scores were described as previously reported^34^.

### Immunohistochemistry

For the immunohistochemistry assays, the colon tissues were dissected, fixed in 4% paraformaldehyde for 12 h at 4 °C, dehydrated overnight with 30% sucrose at 4 °C, embedded in optimal cutting temperature compound and immediately frozen at − 80 °C. Samples sectioned at a thickness of 10 μm were washed with PBS and permeabilization solution with 0.5% Triton X-100 in turn. Samples were then blocked in 3% horse serum in PBS for 30 min and incubated sequentially with the following primary antibodies: anti-collagen I (Cat. ab260043, Abcam Ltd., UK). After incubation with corresponding secondary antibodies (Cat. A11006, Invitrogen, Carlsbad, CA, USA) at room temperature for 1 h, sample sections were treated with a peroxidase substrate DAB kit (Cat. ab64238, Abcam Ltd., UK) and counterstained with hematoxylin.

### 16S rRNA gene sequencing

Total bacterial genomic DNA of fecal samples was extracted using a rapid DNA spin extraction kit (Cat. 117033600, MP Biomedicals, Santa Ana, CA, USA) in accordance with the manufacturer’s instructions. The DNA content was then detected by agarose gel electrophoresis and Nanodrop method (Thermo Scientific, NC2000). The DNA was amplified with specific bacterial primers targeting the 16S rRNA gene containing the V3-V4 region using universal primers 338F (5’-ACTCCTACGGGAGGCAGCA-3’) and 806R (5’-GGACTACHVGGGTWTCTAAT-3’). PCR amplification was purified using Agencourt AMPure Beads (Beckman Coulter, Indianapolis, IN). The PCR amplification products were quantified using the fluorescence reagent Quant-iT PicoGreen dsDNA Assay Kit (Cat. P11496, Invitrogen, Carlsand, CA, USA) and a quantitative microplate reader (BioTek, FLx800). Sequencing was carried out using the Illumina MiSeq platform and MiSeq-pe250 in Shanghai Pesino Biotechnology Co., Ltd (Shanghai, China). All sample sequences were clustered according to the distance between the sequences using Uparse software (v7. 0. 1001), and were divided into operational classification units (OTUs) by plotting the relationship graph between the changes in OTUs and the clustering similarity value. A 97% similarity value was selected for OTU analysis and taxonomic analysis. QIIME 2.0 was then applied to calculate the α-diversity index, including the Chao1 index, Shannon index and Simpson index. UniFrac distance measurement and principal coordinate analysis (PCoA) were selected for β-diversity analysis of the gut microbiota structure, and the gplots package from R software (R 3.4.0) was used for heatmap analysis. Linear discriminant analysis (LDA) of the effect size (LEfSe) was performed with LEfSe software (Version 1.0) and applied to calculate the OTU abundance to determine the differences between groups. Heatmap analysis was performed using R Statistical Software (R version 3.4.0: Foundation for Statistical Computing, Vienna, Austria).

### Statistical analysis

First, the normality of the distribution of dates was tested using the Shapiro–Wilk normality test. Second, statistical comparisons of different groups were evaluated by unpaired Student's t test or one-way ANOVA followed by Tukey’s multiple comparisons. If the data were nonnormally distributed, the Kruskal–Wallis test followed by the Mann–Whitney U test was used. Spearman’s correlation analysis was performed using R Statistical Software (R version 3.4.0: Foundation for Statistical Computing, Vienna, Austria)^35^, and a clustering heatmap of correlation coefficients was calculated by Ward's hierarchy. GraphPad Prism 6.00 (GraphPad Prism Software, San Diego, California, USA, www.graphpad.com) software was used for statis-tical analysis. All values were expressed as the means ± standard deviation (SD). A value of p < 0.05 was considered statistically significant.

## Results

### Rutin improved glucose and lipids metabolism in diabetic mice

**Figure 1:**
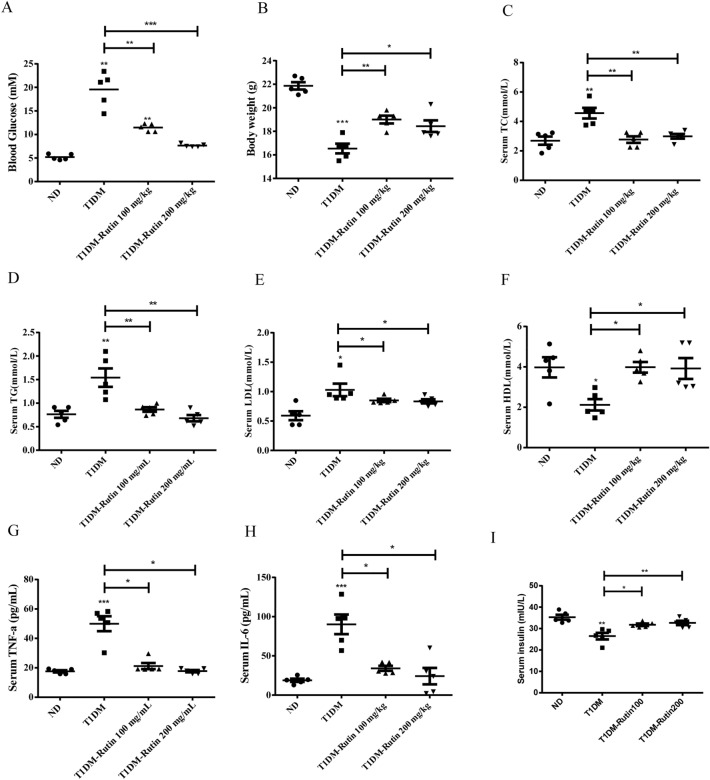
Effects of rutin on serum glucose and lipids in STZ-induced diabetic mice. (**A**) Fasting serum glucose. (**B–I**) Serum TC, TG, LDL-C, HDL-C, TNF-α, IL-6, and insulin levels. Values are means ± SD (n = 5): *p < 0.05, **p < 0.01, ***p < 0.001.

**Figure 2:**
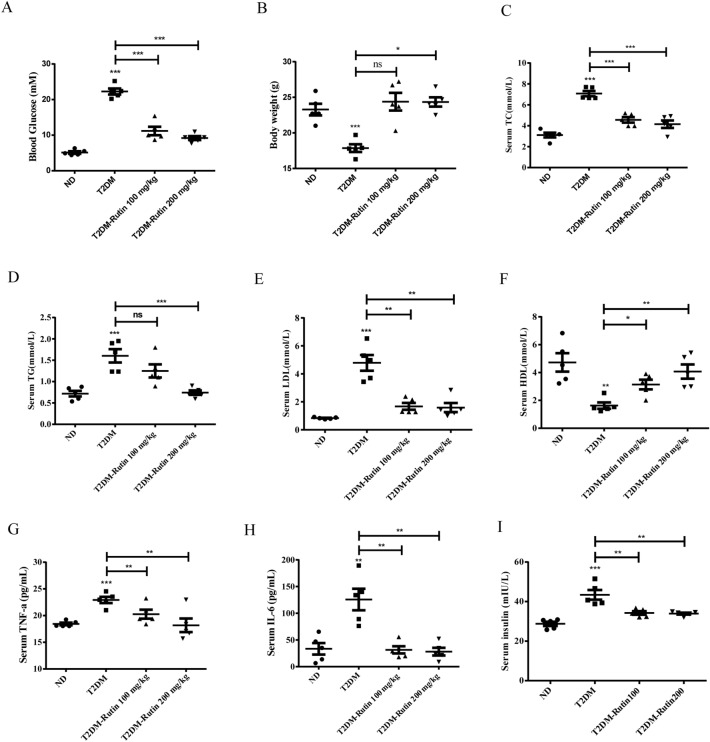
Effects of rutin on serum glucose and lipids in HFD/STZ-induced diabetic mice. (**A**) Fasting serum glucose. (**B–I**) Serum TC, TG, LDL-C, HDL-C, TNF-α, IL-6, and insulin levels. Values are means ± SD (n = 5): *p < 0.05, **p < 0.01, ***p < 0.001.

To investigate the effect of rutin on glucose and lipids in diabetic mice, the STZ-induced T1DM mouse model and HFD/STZ-induced T2DM mouse model were successfully established, followed by administration of 100 or 200 mg rutin per kg body weight for 4 weeks. The metabolic performances of diabetic mice showed that T1DM mice featured decreased body weights and HDL-C levels, increased fasting serum glucose, increased concentrations of TC, TG, and LDL-C, increased secretion of proinflammatory cytokines TNF-α and IL-6, and less secretion of fasting insulin (Fig. 1, Supplementary Table 1) compared with normal mice. Similar metabolic performances occurred in T2DM mice except for fasting serum insulin, which was upregulated in T2DM mice (Fig. 2, Supplementary Table 2). However, rutin alleviated the symptoms that are typical disorders related to glucose and lipid metabolism in T1DM and T2DM mice. Collectively, these observations suggest that rutin could effectively improve glucose and lipids metabolism in diabetic mice.

### Rutin alleviated colon lesions in diabetic mice

**Figure 3:**
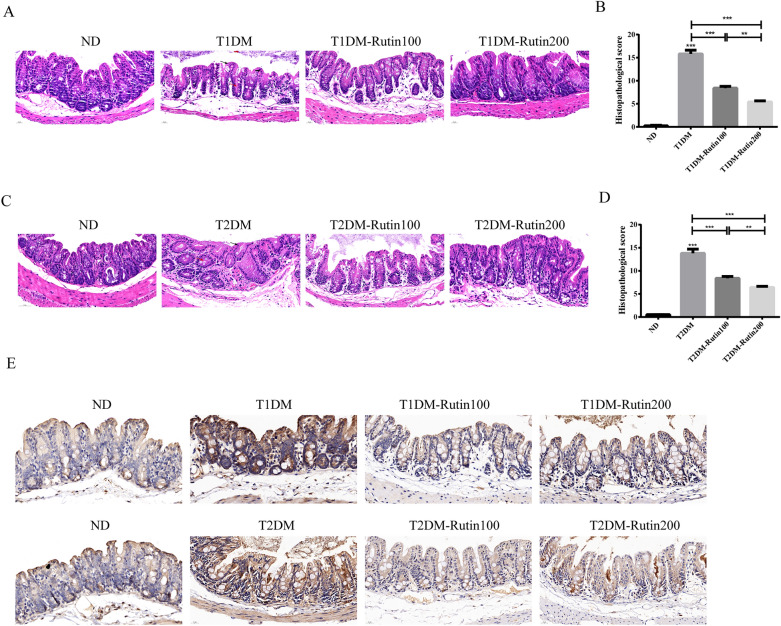
Rutin improved colon lesions in diabetic mice. (**A**) H&E staining of colon tissues in ND, T1DM, T1DM-Rutin100 and T1DM-Rutin200 mouse groups (scale bar: 20 μm). Red arrows: crypts; black arrows: villi. (**B**) Histopathological scores of colon tissues in T1DM mice using H&E staining. (**C**) H&E staining of colon tissues in ND, T2DM, T2DM-Rutin100 and T2DM-Rutin200 groups. Red arrows: vacuole; black arrows: villi (scale bar: 20 μm). (**D**) Histopathological scores of colon tissues in T2DM mice by H&E staining. (**E**) Immunohistochemistry indicated that collagen I protein levels were increased in diabetic mice, which was recovered by rutin treatment (scale bar: 20 μm). Values are means ± SD (n = 5): *p < 0.05, **p < 0.01, ***p < 0.001.

To investigate the effect of rutin on colonic lesions in diabetic mice, colon tissues were stained with H&E. Figure 3 and Supplementary Table 3 show that the villi of colon tissues in the T1DM and T2DM groups were arranged in a disorderly manner and were easy to break, along with many vacuolar changes and damaged crypts, compared to those in the ND group. Treatment with rutin exhibited significant protection against diabetes-related colon lesions. In addition, the collagen I protein level was increased in diabetic mice, which was reversed by rutin treatment. These results indicated that rutin could ameliorate the progression of intestinal fibrosis diseases in diabetic mice. Furthermore, the greater the amount of rutin used, the better the colon lesions were improved.

### Rutin changed the overall community structure of the gut microbiota in diabetic mice

**Figure 4:**
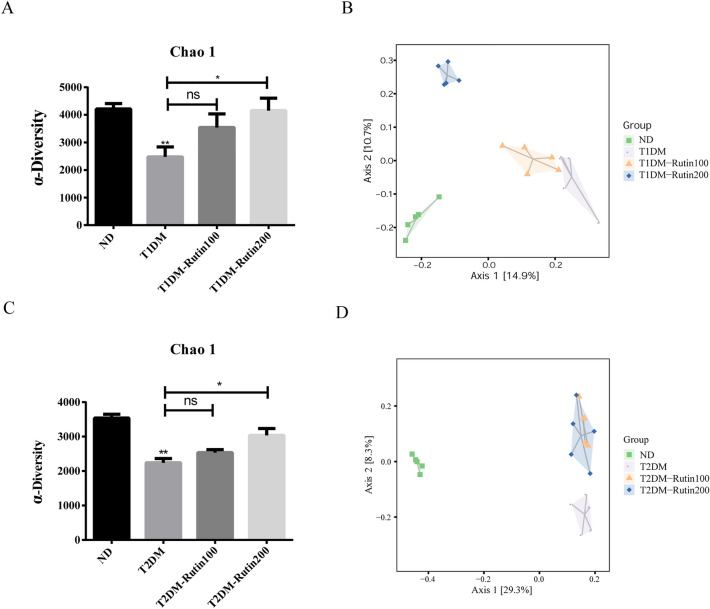
Rutin altered the gut microbiota diversity in T1DM and T2DM mice. (**A**) The Chao 1 index in the α-diversity analysis in T1DM mice. (**B**) β-diversity analysis in the ND, T1DM, T1DM-Rutin100, and T1DM-Rutin200 groups. (**C**) The Chao 1 index in α-diversity analysis in T2DM mice. (**D**) The β-diversity analysis in the ND, T2DM, T2DM-Rutin150, and T2DM-Rutin200 groups. One-way ANOVA was used for statistical comparisons of different groups. Values are means ± SD (n = 5): *p < 0.05, **p < 0.01, ***p < 0.001.

The V3-V4 region of bacterial 16S rRNA in fecal samples from the experimental mice was classified and sequenced to explore the effects of rutin on the overall community structure of gut microbiota in diabetic mice. For the experiment with T1DM mice, a total of 2,672,620 original sequences were obtained and an average of 127,267 ± 17,666 sequences were acquired for each sample. After quality screening, 2,119,396 sequences were generated with an average of 100,923 ± 13,879 sequences for each fecal sample. The abundance grade curves and species accumulation curves indicated that most of the gut microbial diversity was captured with the current sequencing depth in each sample (Fig. S1). Based on the α-diversity analysis, the results showed that the Chao1, Shannon and Simpson indexes reflected both the abundance and diversity of microbial communities. The Chao1 index was significantly downregulated in T1DM mice. This change was reversed by rutin treatment (Fig. 4A and Supplementary Table 4). The Simpson and Shannon indexes were not significantly different among the groups (Fig. S2 and Supplementary Table 5). According to the principal coordinate analysis (PCoA) of weighted UniFrac and unweighted UniFrac based on the β-diversity analysis, it was revealed that gut microbiota in the ND group, T1DM group, T1DM-Rutin100 group and T1DM-Rutin200 group were clearly distinguished, suggesting that there were different microbiota structures among those groups (Fig. 4B, Fig. S3, Supplementary Table 6). In T2DM mice, a total of 2,017,480 original sequences were obtained, and an average of 106,183 ± 6479 sequences were acquired for each sample. A total of 1,562,265 effective sequences were generated with an average of 82,224 ± 499 sequences for each sample after quality screening. The abundance grade curves and species accumulation curves were drawn as shown in Fig. S4. The α-diversity analysis showed that the Chao1 index was significantly decreased in the T2DM group, indicating lower gut microbiota diversity in this group, which was reversed by rutin (Fig. 4C and Supplementary Table 7). No significant difference in the Simpson and Shannon indexes were observed among the different groups (Fig. S5 and Supplementary Table 8). The β-diversity calculated by unweighted UniFrac distances showed that the gut microbiota was clearly distinguished in the ND, T2DM, T2DM-Rutin100, and T2DM-Rutin200 groups, suggesting different microbiota among those four groups (Fig. 4D, Fig. S6, and Supplementary Table 9). The results showed that rutin changed the overall community structure of the gut microbiota in both T1DM and T2DM mice.

### Rutin regulated the composition of the gut microbiota in diabetic mice

**Figure 5:**
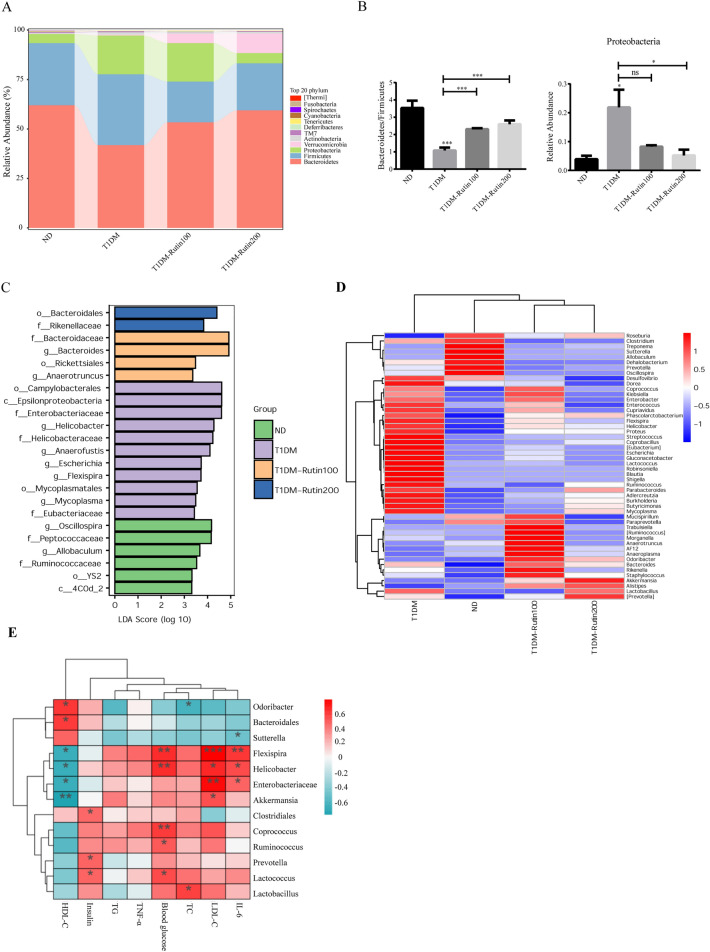
Effects of rutin on the gut microbiota composition in T1DM mice. (**A**) The relative abundances of gut microbiota constituents at the phylum level. (**B**) *Bacteroidetes*/*Firmicutes* in feces of mice and relative abundance of *Proteobacteria* at the phylum level. (**C**) LEfSe analysis of gut microbiota in the ND, T1DM, T1DM-Rutin100, and T1DM-Rutin200 groups. LDA scores > 2 and P < 0. 05 are the taxonomic groups with significant differences between groups. (**D**) Heatmap of the relative abundance of gut microbiota at the genus level. Each column in the plot represents a sample, and each row represents the community structure. The color represents the relative abundance of the species. The heatmap was created using Sangerbox 3.0 (http://vip.sangerbox.com/). (**E**): Spearman’s correlation analysis of gut microbiota and metabolic parameters. Values are means ± SD (n = 5): *p < 0.05, **p < 0.01.

To study whether rutin regulates the composition of the gut microbiota in diabetic mice. We performed 16S rRNA sequencing for each mouse fecal sample. *Firmicutes* and *Bacteroidetes* at phylum level accounted for approximately 82.5% in the T1DM group (Fig. 5A and Supplementary table 10). Our data showed that the *Bacteroidetes*/*Firmicutes* (B/F) ratio in T1DM group was significantly lower than that in the ND group, but the B/F ratios in both the T1DM-Rutin100 group and T1DM-Rutin200 were higher than in the T1DM group. Compared to ND group, the relative abundance of *Proteobacteria* was significantly increased in the T1DM group, which was significantly reversed by rutin (Fig. 5B). LEfSe analysis was conducted to identify differentially abundant bacterial taxa among the 4 groups (LDA score > 2) (Fig. 5C and Supplementary Table 11). T1DM showed selective enrichment in 11 communities including f_*Enterobacteriaceae*, g_*Helicobacter*, g_*Escherichia*, and g_*Mycoplasma*, while T1DM-Rutin100 showed selective enrichment in 4 communities, including f_*Bacteroidaceae*, g_*Bacteroides*, o_*Rickettsiales* and g_*Anaerotruncus*. Moreover, two microbial communities were enriched in the TIDM-Rutin200 group, including o_*Bacteroidales* and f_*Rikenellaceae*. At the order level, the relative abundances of *Lactobacillales*, *Coriobacteriales*, and *Campylobacterales* were increased in the T1DM group, but decreased after rutin treatment. Furthermore, the relative abundances of *Bacteroidales* and *Verrucomicrobiales* were increased by rutin (Fig. S7 and Supplementary Table 12). The heatmap clustering analysis showed that compared to the ND group, the relative abundances of *Dorea*, *Coprococcus*, *Enterobacter*, *Flexispira*, *Helicobacter*, *Proteus*, *Streptococcus*, *Coprobacillus*, *Escherichia* and *Lactococcus* were increased in the T1DM group, which was reversed by rutin. Furthermore, rutin increased the relative abundances of *Odoribacter*, *Rikenella*, *Staphylococcus*, *Akkermansia*, *Alisipes*, *Lactobacillus*, *Prevotella* and *Roseburia*. These bacteria were negatively associated with diabetes (Fig. 5D). Additionally, Spearman’s correlation analysis showed that the gut microbiota was correlated with metabolic parameters related to diabetes (Fig. 5E and Supplementary Table 13). Serum glucose was positively correlated with the relative abundance of *Flexispira* and *Helicobacter*. Serum TC, TG, LDL-C, TNF-α, and IL-6 levels were positively correlated with the relative abundances of *Enterobacteriaceae*, *Lactobacillus*, and *Lactococcus*. The abundance of *Sutterella* was negatively associated with insulin.

**Figure 6:**
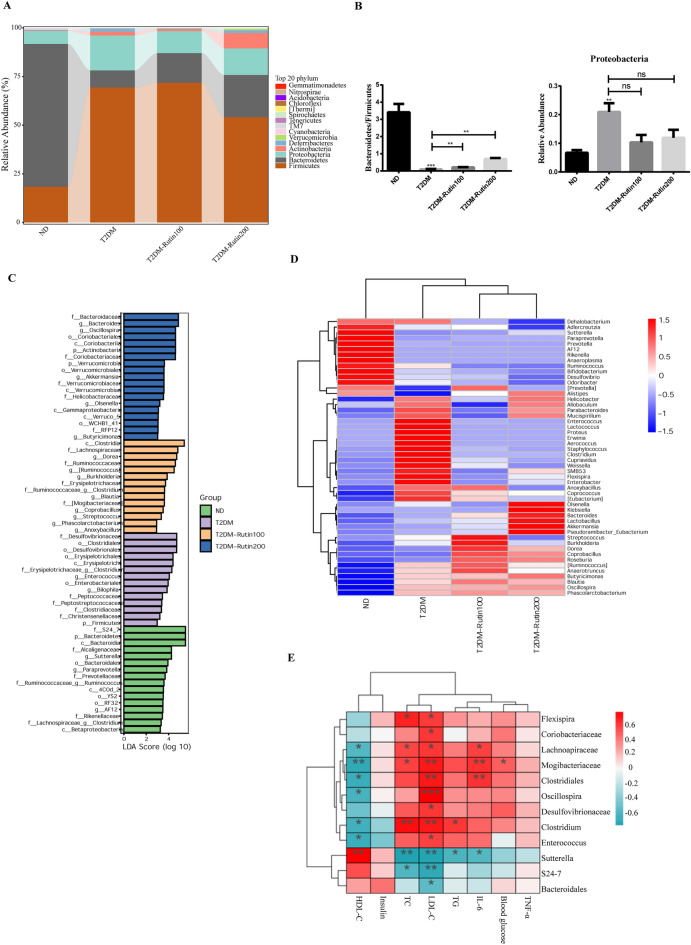
Effects of rutin on the gut microbiota composition in T2DM mice. (**A**) The relative abundances of gut microbiota constituents at the phylum level. (**B**) *Bacteroidetes*/*Firmicutes* in feces of mice and relative abundance of *Proteobacteria* at the phylum level. (**C**) LEfSe analysis of gut microbiota in the ND, T2DM, T2DM-Rutin100, and T2DM-Rutin200 groups, LDA scores > 2 and P < 0. 05 are the taxonomic groups with significant differences between groups. (**D**) Heatmap of the relative abundance of gut microbiota at the genus level. Each column in the plot represents a sample, and each row represents the community structure. The color represents the relative abundance of the species. The heatmap was created using Sangerbox 3.0 (http://vip.sangerbox.com/). (**E**): Spearman’s correlation analysis of gut microbiota and metabolic parameters. Values are means ± SD (n = 5): *p < 0.05, **p < 0.01.

In the experiments with T2DM mice, *Firmicutes* and *Bacteroidetes* at the phylum level accounted for approximately 83% in the T2DM group (Fig. 6A and Supplementary table 14). The B/F ratio in T2DM group was lower than that in the ND group, however, rutin significantly reversed this change. The relative abundance of *Proteobacteria* in the T2DM group was significantly higher than ND group, and rutin treatment significantly decreased the relative abundance of *Proteobacteria* (Fig. 6B). Furthermore, the LEfSe analysis showed that T2DM had selective enrichment in 14 communities, including f_*Desulfovibrionaceae*, o_*Clostridiales*, c_*Erysipelotrichi*, g_*Bilophila* and p_*Firmicutes*, while the T2DM-Rutin100 group had selective enrichment in 14 communities including f_*Lachnospiraceae*, g_*Blautia*, g_*Anoxybacillus* and g_*Coprobacillus*. However, 19 communities were selectively enriched in the T2DM-Rutin200 group, such as g_*Bacteroides*, g_*Oscillospira*, p_*Verrucomicrobia*, g_*Akkermansia* and g_*Butyricimonas* (Fig. 6C and Supplementary Table 15). Rutin decreased the upregulated relative abundances of *Erysipelotrichales*, *Lactobacillales* and *Bacillales* in T2DM mice at the order level but increased those of *Bacteroidales*, *Burkholderiales* and *Coriobacteriales* (Fig. S8). The relative abundances of *Helicobacter*, *Allobaculum*, *Parabacteroides*, *Mucispirillum*, E*nterococcus*, *Lactococcus*, *Proteus*, *Staphylococcus* and *Enterobacter* were upregulated in T2DM mice at the genus level, which were reversed by rutin treatment. Additionally, rutin increased the relative abundances of *Klebsiella*, *Bacteroides*, *Lactobacillus*, *Akkermansia*, *Pseudoramibacter*, *Eubacterium*, *Roseburia*, *Butyricimonas* and *Alisipes* (Fig. 6D and Supplementary Table 16). The serum glucose level was positively correlated with the relative abundance of *Mogibacteriaceae*. The relative abundances of *Clostridiales*, *Mogibacteriaceae*, and *Lachnospiraceae* were positively correlated with LDL-C, and IL-6 levels, while those of *Mogibacteriaceae* and *Lachnospiraceae* were negatively associated with HDL-C (Fig. 6E and Supplementary Table 17). These findings indicated that rutin could regulate the composition of the gut microbiota in both T1DM and T2DM mice.

## Discussion

Accumulating evidence suggests that the gut microbiota plays an important role in regulating obesity, diabetes, colon cancer and nonalcoholic fatty liver disease^8^. Rutin is a type of flavonoid glycoside enriched in tea and buckwheat that has multiple biological activities, such as antioxidant, anti-inflammatory, antidiabetic and free radical scavenging activities. A previous study suggested that the combination of rutin and vitamin C can effectively improve oxidative stress and lower serum glucose in patients with T2DM^22^. Rutin can not only prevent STZ-induced oxidative damage, but also protect islet β cells to increase insulin secretion and therefore lower serum glucose^36^. However, whether rutin alleviates colon lesions and regulates the gut microbiota in diabetic mice is entirely unknown. Our study found that rutin could significantly improve the levels of serum glucose, TC, TG, and LDL-C in diabetic mice and increase HDL-C levels. Chronic inflammation caused by high serum glucose and excessive visceral fat accumulation could increase the risk of many different cancers, such as colon cancer and liver cancer^37^. We found that the villi of colon tissues were arranged disorderly and easy to break, along with many vacuolar changes and damaged crypts in diabetic mice, this phenomenon is might strongly associated with the loss of tight junction proteins, which are the expression levels of tight junction proteins in patients with diabetes are abnormal, which play an important role in maintaining the structure of colon^38^, however, whether rutin affects the expression levels of tight junction proteins in colon tissue of diabetic mice, we will further explore this hypothesis in the future. Disorders of the gut microbiota were reported to be closely related to the occurrence and development of colonic diseases^39,40^. We determined the effects of rutin on gut microbiota dysbiosis and colon lesions in diabetic mice and found that rutin significantly alleviated colon lesions in T1DM and T2DM mice (Fig. S9).

It is known that the gut microbiota condition affects the pathophysiological states of metabolic diseases. The abnormal gut microbiota in diabetic mice might be related to the occurrence of obesity, diabetes, inflammation and pathological microenvironments^41,42^. Reduced bacterial function diversity and low community stability were characterized in diabetic mice^43^. We hypothesized that the antidiabetic effects of rutin and its ability to repair colon lesion damage in diabetic mice might be closely related to the regulation of the gut microbiota. In our study, the abundance grade curves and species accumulation curves indicated that most of the gut microbial diversity was captured with the current sequencing depth in each sample. However, the rank abundance curve estimate for diversity might not be fully representative of reality. In addition, Chao1 and Shannon indexes were higher in the rutin treatment groups than in the T1DM, or T2DM group. Diversity analysis showed differences in gut microbiota distribution among the groups. However, there was still a difference between the intestinal flora after rutin treatment and that of nondiabetic mice. It is possible that the dosage of rutin is not enough to achieve the therapeutic effect, which might have an auxiliary therapeutic effect. The above results showed that rutin significantly changed the structure of the intestinal flora in diabetic mice, indicating a novel pathway through which rutin alleviates diabetes-related symptoms. It has been reported that obesity and metabolic syndromes are associated with changes in gut microbiota composition, including a decreased *Bacteroidetes/Firmicutes* (B/F) ratio and relative abundance of *Proteobacteria*^44^. It was also found that the abundance of *Firmicutes* and *Bacteroidetes* in diabetic mice was related to hyperglycemia and inflammatory state^45^. In accordance with previous studies, our results showed that rutin effectively increased *Bacteroidetes* and decreased *Firmicutes*, along with an increased ratio of *Bacteroidetes* to *Firmicutes*. The increase in the abundance of *Proteobacteria* could promote the endotoxin content, thus leading to the occurrence and development of chronic inflammatory diseases^46^. We also demonstrated that rutin reversed the relative abundance of *Proteobacteria* in diabetic mice. Furthermore, previous studies suggested that *Erysipelotrichales* were correlated with the development of obesity, systemic inflammation and metabolic diseases^47,48^. In our study, the *Erysipelotrichaeae* abundance in T2DM mice was much higher than that in the control ND group, which was reversed by rutin. Cani et al. reported a positive relationship between *Escherichia* and the occurrence and development of diabetes and obesity^49^, whereas rutin reduced the presence of *Helicobacter*, *Enterobacter*, and *Escherichia* in diabetic mice. The increase in the F/B ratio was related to obesity induced by a high-fat diet in mice^50^. Rutin could increase the B/F ratio and reduce the content of *Proteobacteria*, which was closely related to inflammation.

It was reported that *Akkermansia* was a mucin-degrading bacterium and improved insulin resistance^51^. Our data showed that rutin increased *Akkermansia* abundance. The relative abundances of some bacteria, such as *Bilophila* and *Mucispirillum*, showed a significant positive correlation with the development of diabetes^47,52^. Therefore, the protective effect of rutin on diabetic symptoms might be due to the decrease in *Mucispirillum*, *Helicobacter*, *Enterobacter*, *Proteus*, and *Streptococcus* abundances. In addition, rutin increases the relative abundances of bacteria, such as *Odoribacter*, *Rikenella*, *Akkermansia*, *Alistipes* and *Roseburia*, which impairs the development of diabetes. Spearman correlation analysis showed that key communities such as *Enterobacteriaceae*, *Lactobacillus* and *Sutterella* were related to the main metabolic parameters of diabetic mice. It is worth noting that blood lipids and inflammatory factors in diabetes mice are positively correlated with *Lactobacillus*, it is possible that the increase of *Lactobacillus* abundance can protect the damage of inflammation to the body, and may play an important role in immune defenses. *Sutterella* has anti-inflammatory properties and is negatively correlated with the content of IL-6^53,54^. Therefore, the reduction in inflammation in diabetic mice treated with rutin might be due to the increase in *Sutterella* abundance, which was positively related to the decrease in serum IL-6, suggesting that the effect of rutin on the flora might be related to metabolic disorders. Several studies have demonstrated that short-chain fatty acids (SCFAs) are the main metabolites of gut microbiota that play a critical role in regulating metabolic syndrome and maintaining energy homeostasis and host insulin sensitivity^55,56^. Short chain fatty acids such as acetic acid, propionic acid and butyric acid play an important role in improving chronic inflammatory diseases and promoting colon cell health^57^. *Alisipes*, *Roseburia*, *Rikenella* and *Odoribactor* are bacteria that produce butyric acid, propionic acid and butyric acid in the intestine^58,59^. SCFAs produced by these microorganisms can provide energy for the host, improve the acidic environment in the colon, inhibit the production of inflammatory factors, and finally repair mucositis. Our study found that rutin significantly increased the abundances of *Alisipes*, *Odoribacter*, *Roseburia*, and *Rikenella*, which might lead to increased production of SCFAs that act beneficially on colon villi in our experimental diabetic mice. The recovery of colon damage by rutin might therefore be due to the enrichment of SCFA-producing bacteria. Targeted recovery of these SCFA products will provide a new therapeutic approach for diabetes patients.

## Conclusions

In conclusion, rutin exhibited an antidiabetic effect and improved colon lesions in diabetic mice, possibly by regulating the gut microbiota community structure and composition, which might be a potential new strategy for treating diabetic patients, protecting intestinal health and/or preventing colon cancer carcinogenesis. Further study is needed to reveal the detailed mechanisms through which rutin regulates the gut microbiota in diabetic mice.

## Supplementary Information

Supplementary Figures.

Supplementary Tables.

## Data Availability

The datasets generated and/or analysed during the current study are available in the Zenodo repository, https://zenodo.org/record/7404629, doi: 10.5281/zenodo.7404629.
