# Supplementary material for: Rutin alleviates colon lesions and regulates gut microbiota in diabetic mice
Source: Sci Rep. 2023 Mar 25;13:4897. doi: 10.1038/s41598-023-31647-z (PMC10039872; doi:10.1038/s41598-023-31647-z)
Supplement: Supplementary file 1 — Supplementary Figures. [file 41598_2023_31647_MOESM1_ESM.pdf]

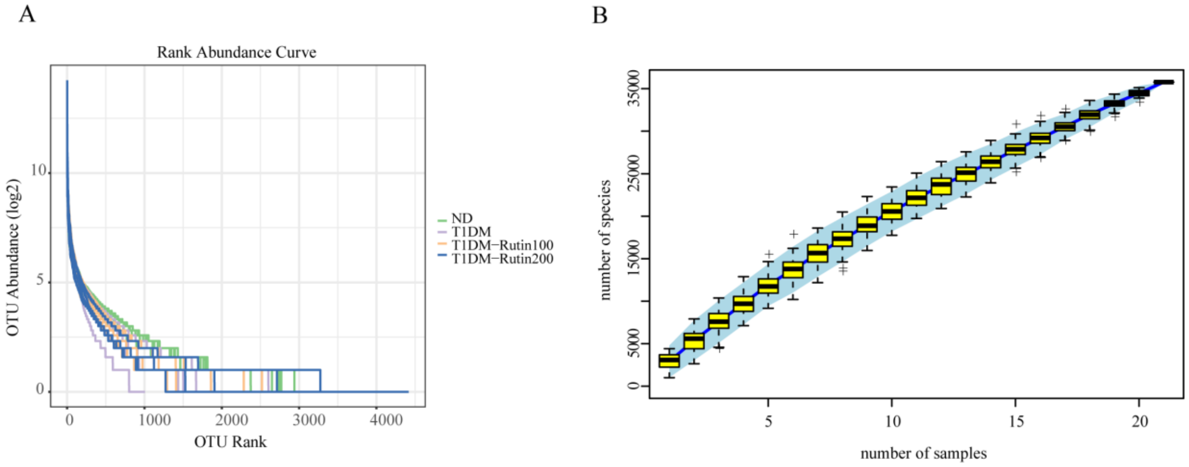

Figure S1. Rank abundance curve and Species accumulation curves in T1DM mice.

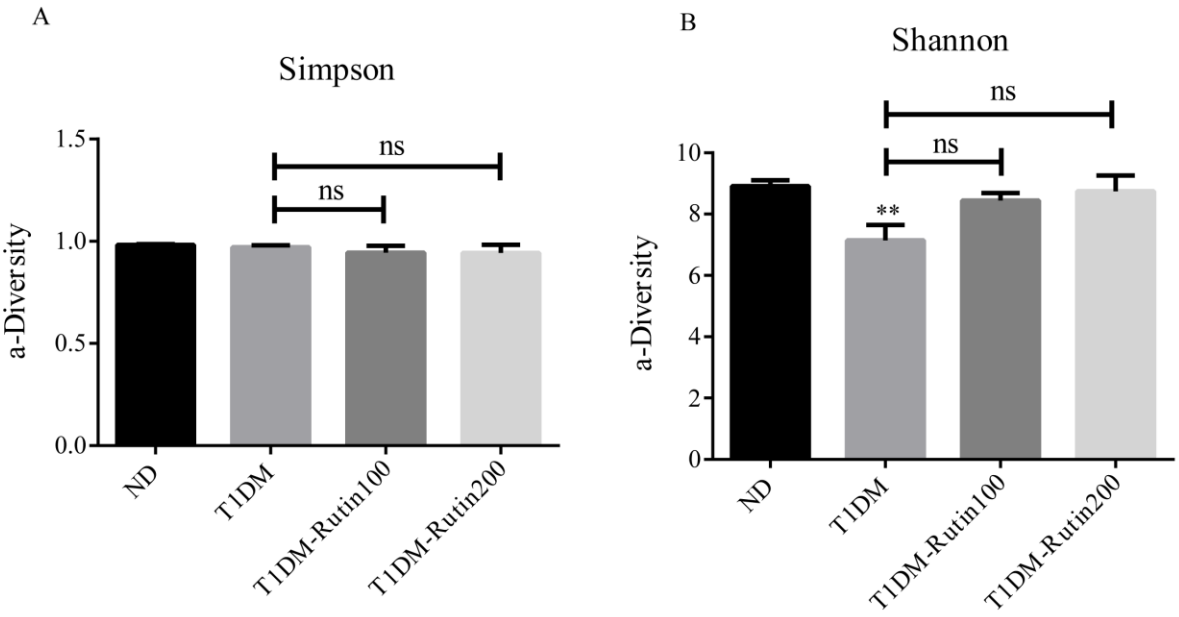

Figure S2. Simpson and Shannon indexes in T1DM mice. A: Simpson index. B: Shannon index.

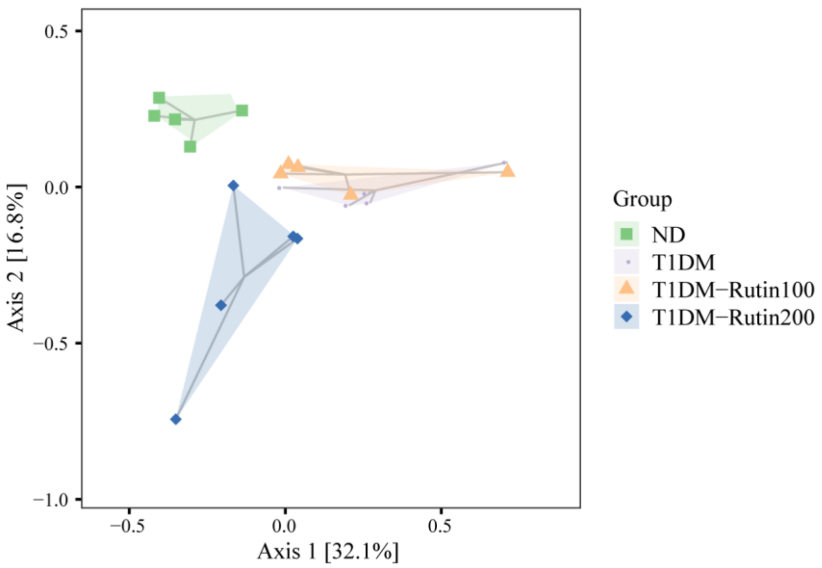

Figure S3. The unweighted UniFrac based on the β-diversity analysis in the ND, T1DM, T1DM-Rutin100, T1DM-Rutin200 groups.

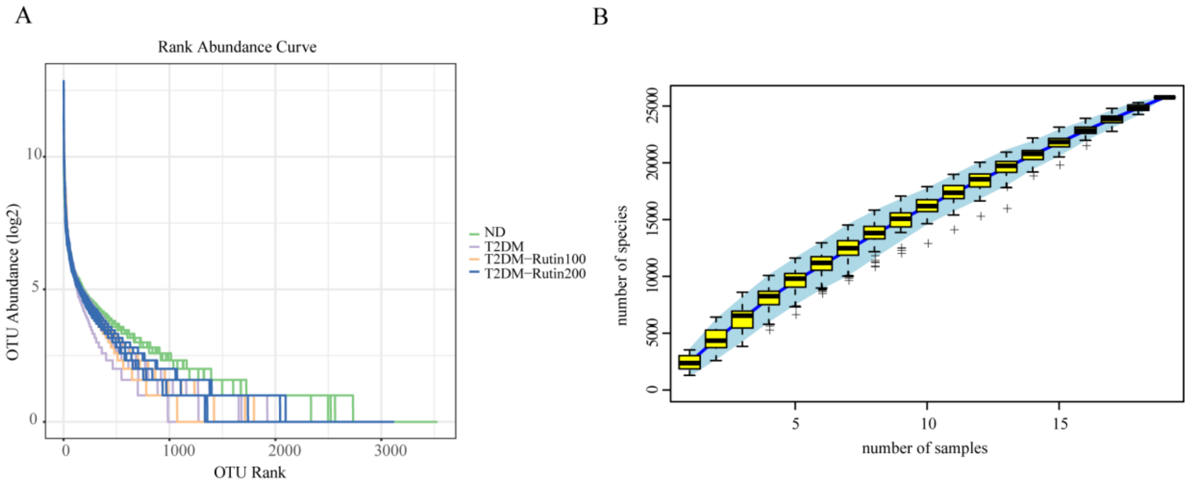

Figure S4. Rank abundance curve and Species accumulation curves in T2DM mice. A: Rank abundance curve. B: Species accumulation curves.

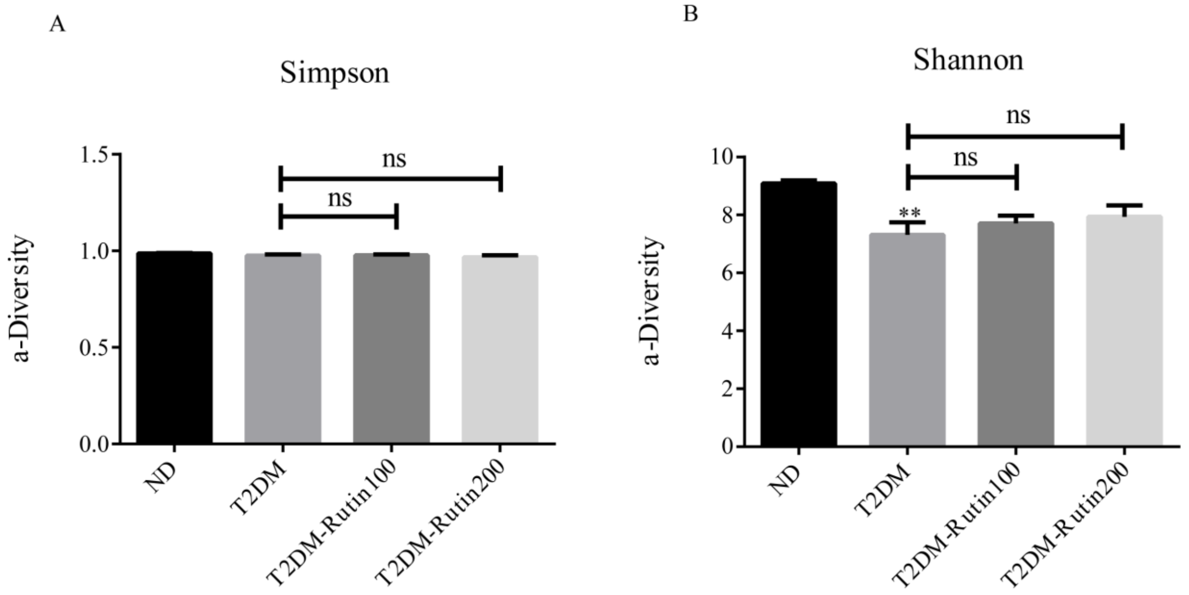

Figure S5. Simpson and Shannon indexes in T2DM mice. A: Simpson index. B: Shannon index.

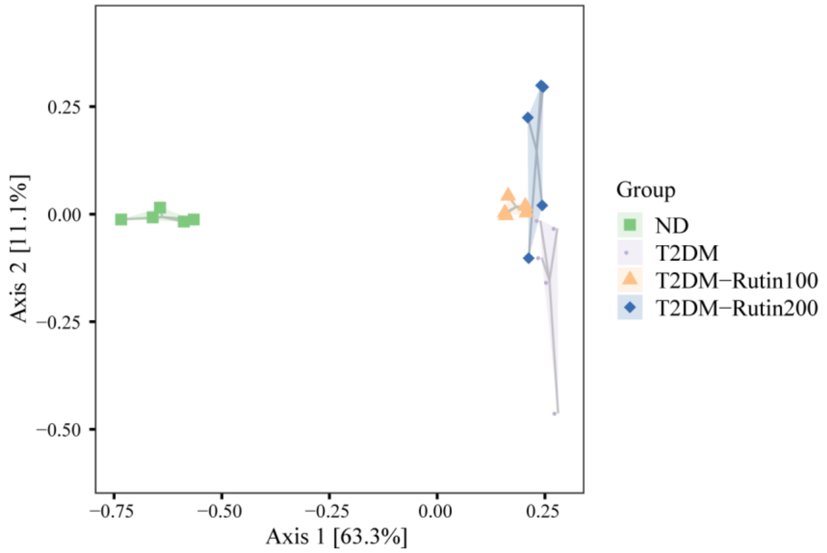

Figure S6. The unweighted UniFrac based on the β-diversity analysis in the ND, T2DM, T2DM-Rutin100, T2DM-Rutin200 groups.

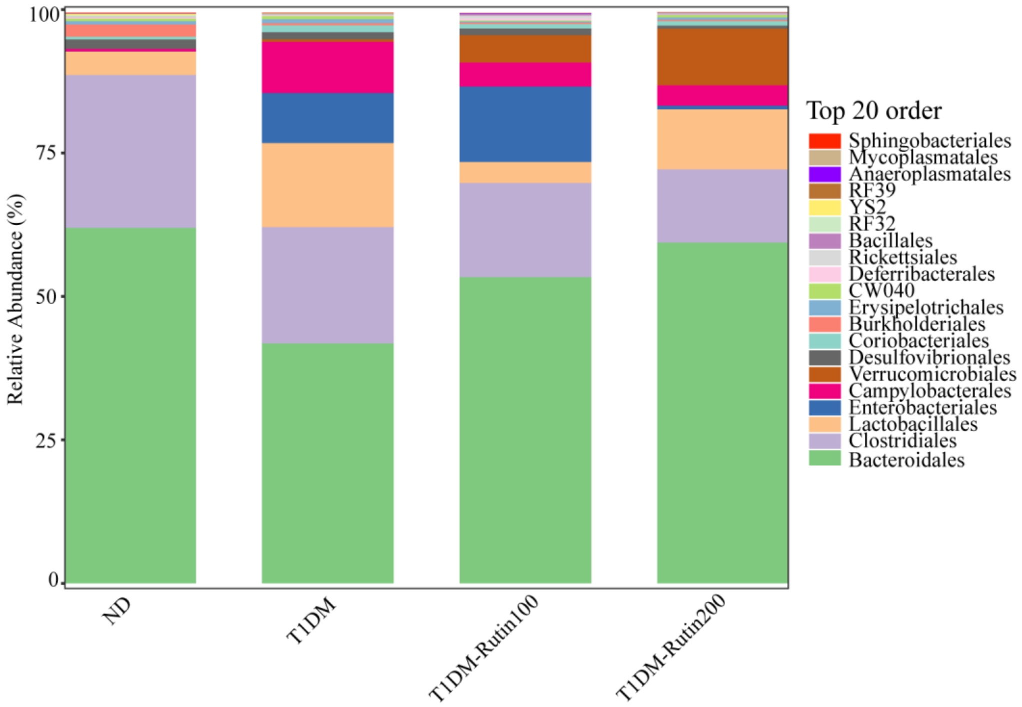

Figure S7. Bacterial taxonomic profiling of gut microbiota at the order level in T1DM mice.

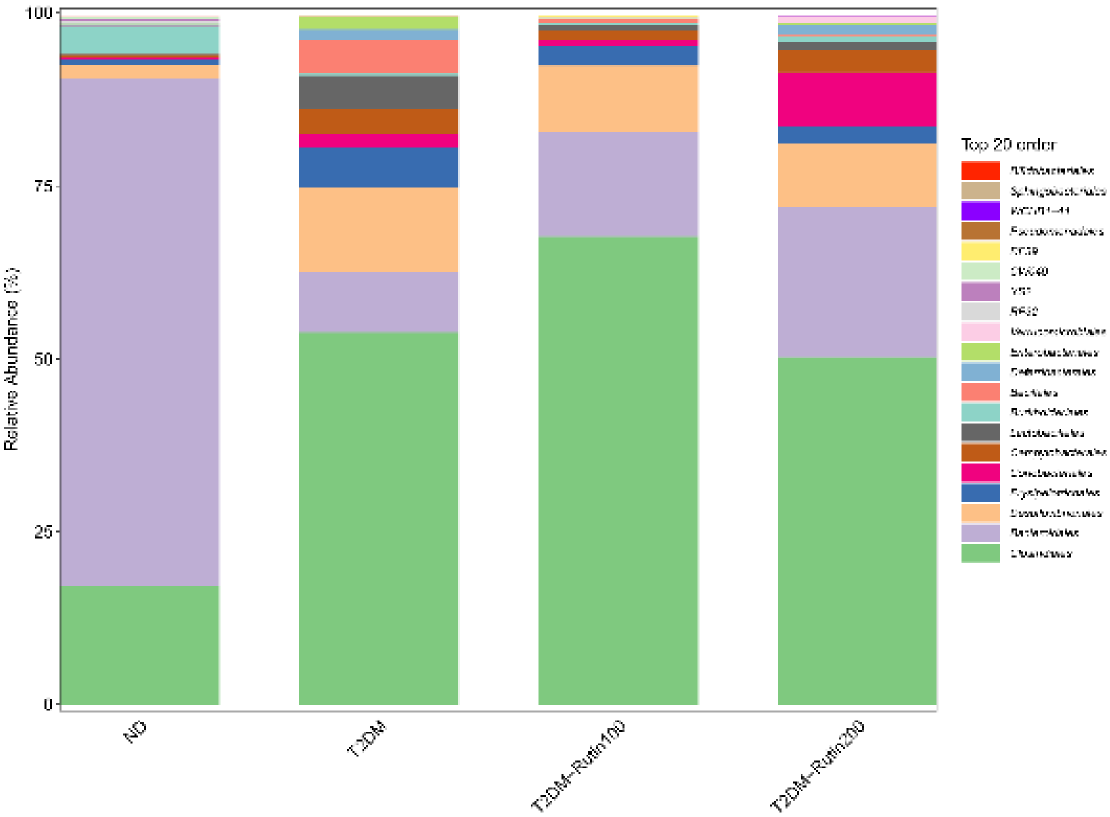

Figure S8. Bacterial taxonomic profiling of gut microbiota at the order level in T2DM mice.

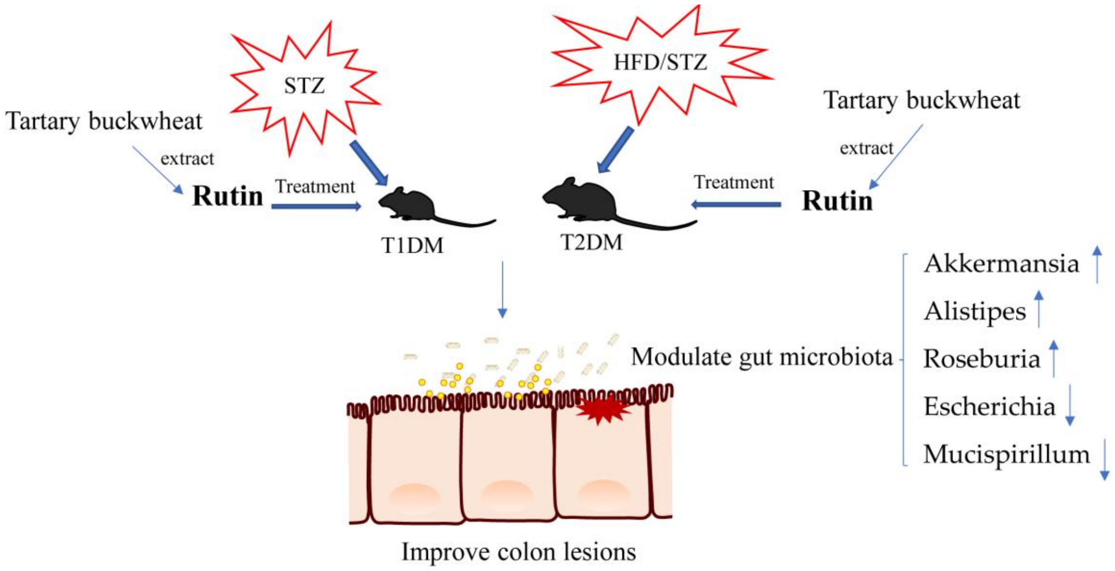

Figure S9. A visual depiction of the experimental design.
